# Impact of iron chelation therapy on mitochondrial function, vascular integrity and inflammation in transfusion-dependent myelodysplastic syndromes

**DOI:** 10.3389/fimmu.2025.1683941

**Published:** 2025-11-10

**Authors:** Regina García-Delgado, Gloria Moreno-Carrasco, Manuel Carrasco-Gomariz, Silvia García-Segovia, Rodolfo Matías Ortiz Flores, Julio Torres-González, Gonzalo Gallego-Fuillerat, Juan Antonio López-Villodres, Alejandro Escamilla-Sánchez

**Affiliations:** 1 Management Unit (UGC) Hematología y Hemoterapia, Hospital Universitario Virgen de la Victoria, Málaga, Spain; 2 BE21-Hematología e Inmunoterapia, Instituto de Investigación Biomédica de Málaga y Plataforma en Nanomedicina (IBIMA Plataforma BIONAND), Málaga, Spain; 3 Departamento de Fisiología Humana, Histología Humana, Anatomía Patológica y Educación Físico-Deportiva, Facultad de Medicina, Universidad de Málaga, Málaga, Spain; 4 UGC Farmacia, Hospital Universitario Virgen de la Victoria, Málaga, Spain

**Keywords:** iron chelation, deferasirox, myelodysplastic syndrome, oxidative stress, endothelial function, cytokines, ferritin

## Abstract

**Background:**

Patients with myelodysplastic syndromes (MDS) frequently develop chronic transfusion dependence, leading to progressive iron overload. This accumulation of non-transferrin-bound iron (NTBI) contributes to oxidative stress, mitochondrial dysfunction, endothelial damage, impaired vascular regeneration, and heightened inflammation.

**Objectives:**

To assess whether iron chelation therapy can reverse cellular and vascular injury, redox imbalance, and immune dysfunction in transfusion-dependent MDS patients.

**Methods:**

Peripheral blood from 23 transfusion-dependent MDS patients was analysed in a paired pre-/post-treatment design. Patients received daily oral deferasirox at standard clinical dosing for approximately 6 months (median). Flow cytometry was employed to evaluate reactive oxygen species (ROS), expression of adhesion molecules, mitochondrial membrane potential, circulating endothelial progenitor cells (EPCs), and intracellular levels of key pro-inflammatory cytokines.

**Results:**

Chelation therapy was associated with a ~55% decrease in serum ferritin and robust redox recovery: leukocyte H_2_O_2_ and superoxide decreased ~3.8-fold and ~3.2-fold, respectively (both p<0.0001), intracellular glutathione increased ~3.3-fold (p<0.0001), and mitochondrial membrane potential rose ~2.6-fold (p<0.0001). Endothelial injury and adhesion were attenuated (Annexin V ~2-fold↓; ICAM-1 ~33%↓; VCAM-1 ~15%↓; E-selectin ~25%↓; P-selectin ~52%↓; all p<0.0001), while endothelial progenitors and mature endothelial cells increased ~2.4–2.5-fold (both p<0.0001). Pro-inflammatory cytokines IL-1 (p=0.0013), IL-3, IL-6 and TNF-α (all p<0.0001) decreased, whereas IFN-γ increased (p<0.0001) consistent with attenuation of NF-κB-related inflammatory signaling and partial immune reactivation.

**Conclusions:**

Iron chelation may modulate disease-relevant redox, endothelial, and cytokine pathways in transfusion-dependent MDS, generating mechanistic hypotheses for prospective clinical validation. These findings support the concept that NTBI reduction mitigates pathogenic processes relevant to disease progression, warranting confirmation in prospective studies integrating clinical endpoints.

## 1 Introduction

Myelodysplastic syndromes (MDS) represent a heterogeneous group of clonal hematopoietic disorders characterized by ineffective hematopoiesis, peripheral cytopenia, and a variable risk of progression to AML. Repetitive red blood cell (RBC) transfusions remain a cornerstone of supportive care in transfusion-dependent MDS (1). However, because the human body lacks a regulated mechanism for iron excretion, chronic transfusional dependency leads to progressive iron accumulation (2). Once transferrin saturation is exceeded, excess iron circulates as non–non-transferrin-bound iron (NTBI), a redox-active form capable of catalyzing the Fenton reaction and promoting reactive oxygen species (ROS) generation. Recent studies have provided compelling evidence that iron overload is not merely a passive consequence of supportive care but actively contributes to MDS progression (3, 4). Iron-mediated reactive oxygen species (ROS) production contributes to cellular injury across multiple biological compartments, including hematopoietic progenitors, endothelial cells, and immune effector cells (5). Mitochondrial dysfunction is a significant oxidative stress outcome. Mitochondrial membrane depolarization and oxidative phosphorylation disruption are crucial factors in the failure of blood cell production and abnormal cell growth in MDS (6–8). Mitochondrial glutathione (mGSH) plays a central role in preserving redox equilibrium and mitochondrial function (9). Decreased mGSH levels have been shown to worsen oxidative stress and disrupt mitochondrial polarization. This can result in genomic instability and cellular senescence (10, 11). Beyond its intracellular consequences, iron overload disrupts vascular homeostasis. Endothelial dysfunction, driven by ROS and upregulation of adhesion molecules (e.g., ICAM-1, VCAM-1, and selectins), fosters an inflammatory microenvironment conducive to thrombosis and immune dysregulation (12–14). Circulating endothelial progenitor cells (EPCs), which contribute to vascular repair and angiogenesis, are markedly reduced in MDS and inversely correlate with disease burden and progression risk (15). Furthermore, proinflammatory cytokines, including IL-1, IL-6, IL-3, TNF-α, and interferon-γ (IFN-γ) are frequently elevated in MDS, thereby perpetuating marrow inflammation, impairing hematopoietic differentiation, and fueling clonal selection (16). Iron overload also affects immune regulation. Recent studies have highlighted its role in skewing macrophage polarization toward the proinflammatory M1 phenotype and impairing T cell function, further intensifying the dysregulated immune environment characteristic of MDS. Increased attention has been given to the treatment of hematological malignancies, such as MDS (17), due to the role of endothelial progenitor cells in maintaining vascular balance and serving as indicators of disease progression. Biological differences remain a factor in MDS clinical management. Erythropoiesis-stimulating treatments offer advantages for selected individuals. Those requiring transfusions face a lower likelihood of treatment complications, whereas high-risk subtypes still experience treatment failure. This is demonstrated by the limited effectiveness of lenalidomide in certain MDS subtypes (18, 19). This vascular impairment, along with the combined impact of transfusional iron overload, might further harm the blood-forming niches and the endothelial cells in MDS, emphasizing the importance of iron-targeting interventions. Iron chelation therapy, particularly with deferasirox, has traditionally been used to prevent organ damage induced by iron overload (20–22). Nevertheless, accumulating data suggest that chelation may exert pleiotropic biological effects, including anti-inflammatory, antioxidant, and anti-clonal properties, beyond iron removal. However, comprehensive evaluations integrating mitochondrial function, endothelial activation, oxidative stress, and cytokine signaling in the context of iron chelation remain scarce. We carried out a study which integrated iron reduction *via* chelation treatment with alterations in ROS levels, mitochondrial function, vascular damage, and the profile of pro-inflammatory molecules in PBMCs from patients with MDS. Our findings contribute to a growing body of evidence suggesting that iron overload is a modifiable contributor to disease progression and not merely a bystander in MDS pathophysiology. We aim to support its consideration as a disease-modifying intervention in patients with MDS by elucidating the biological sequelae of iron chelation. This integrative approach will allow us to investigate the coordinated biological impact of iron chelation across vascular, oxidative, and immunologic axes.

## 2 Materials and methods

### 2.1 Study design and patient selection

Peripheral blood samples were obtained from 23 patients diagnosed with transfusion-dependent MDS and treated at the Hospital Virgen de la Victoria (Málaga, Spain) between 2009 and 2011. All diagnoses were established according to the World Health Organization (WHO) 2008 classification and reclassified according to the Revised International Prognostic Scoring System (IPSS-R). Patients meeting the required standards were chosen based on a set of strict criteria, which included: (i) a diagnosis of low-risk MDS, (ii) baseline serum ferritin levels >1000 ng/mL, (iii) a history of having received 20 or more RBC units in the preceding year, (iv) treatment with deferasirox for at least six months, and (v) the absence of active infections, autoimmune disorders, or concurrent treatment with immunomodulatory or hypomethylating agents. Healthy donor samples were processed under identical preanalytical and cytometry conditions as patient samples, serving as a reference to confirm assay validity and consistency. The baseline characteristics of the study population are summarized in **Table 1**.

**Table 1:** Baseline characteristics of the study cohort (n = 23).

Characteristic	Patients with MDS (n = 23)
Age (years), mean (range)	64 (29 - 86)
Sex (Male/Female)	M = 18 / F = 5
IPSS-R Classification	Low (n=7), Intermediate I (n = 16),
Baseline serum ferritin level (ng/mL), mean (range)	2090.87 (1342 - 4850)
Hemoglobin (g/dL), mean (range)	7.72 (6.3 – 8.7)
Leukocytes (10 ^3^)/µL), mean (range)	4.66 (0.8 – 11.84)
Neutrophils (10^3^/µL), mean (range)	2.51 (1.1 – 6.4)
Platelets (10^3^/µL), mean (range)	191 (65-885)

### 2.2 Therapeutic intervention: iron chelation

Patients received deferasirox (Exjade®, Novartis) at standard doses approved by the European Medicines Agency. The median length of treatment was nine months, ranging from 6 to 11 months.

### 2.3 Peripheral blood processing and mononuclear cell isolation

Approximately 20 mL of peripheral blood were drawn into EDTA tubes and processed within two hours of collection. Cells were isolated by density gradient centrifugation with Ficoll-Hypaque (Accuspin, Sigma-Aldrich). In certain cases, CD34+ cells were purified with the AutoMACS automated magnetic cell separation system (Miltenyi Biotec).

### 2.4 Flow cytometry

Multiparametric flow cytometric analyses were performed using a dual-laser FACSCalibur® flow cytometer (Becton Dickinson), and data acquisition was conducted using CellQuest software (Becton Dickinson). Instrument calibration was performed daily using standardized fluorescent calibration beads (e.g., CaliBRITE™, BD) to ensure inter-sample reproducibility. The compensation settings were checked in every one of the conducted experimental runs. A minimum of 10,000 events were recorded per sample. Cellular subpopulations, including neutrophils, monocytes, and lymphocytes, were defined based on their forward and side scatter properties and gating strategies were validated. Analyses were performed using standard gating hierarchies with appropriate isotype/FMO controls. Paired pre- and post-treatment samples were acquired in parallel under identical cytometer settings and compensation. The study focused on functional endpoints (adhesion molecules, Annexin V, oxidative stress, mitochondrial polarization), for which mean fluorescence intensity (MFI) was the appropriate metric. The six different markers described were interrogated through sequential 4-color panels, processed in parallel for each subject’s pre- and post-treatment samples using identical instrument settings and compensation.

### 2.5 Oxidative stress and mitochondrial function

Assessment of oxidative stress biomarkers was carried out in total leukocytes and defined subpopulations. Intracellular hydrogen peroxide (H_2_O_2_) levels were determined using 2',7'-dichlorodihydrofluorescein diacetate (H2DCFDA), while superoxide anion (O_2_^-^) generation was measured using dihydroethidium (DHE). Intracellular glutathione content was quantified using mBCI. Cells were incubated with the respective probes at 37 °C for 30 min in the dark, followed by washing with phosphate-buffered saline and immediate flow cytometric analysis. Mitochondrial membrane potential was evaluated using Rhodamine-123 (Sigma-Aldrich) at a final concentration of 4 ng/mL. Cells were incubated in the dark at 37 °C for 30 min, washed, and resuspended in PBS for analysis.

### 2.6 Adhesion molecules and proinflammatory markers

The expression of adhesion molecules, including ICAM-1, VCAM-1, E-selectin, P-selectin and Annexin V, was assessed using fluorochrome-conjugated monoclonal antibodies (BD Pharmingen and R&D Systems). Appropriate fluorochrome-matched isotype controls were used for all surface marker analyses. Intracellular cytokines, including IL-1, IL-3, IL-6, TNF-α, and IFN-γ, were measured after fixation and permeabilization using fluorescein isothiocyanate (FITC)-conjugated antibodies, following the manufacturer’s protocol.

### 2.7 Quantification of circulating endothelial cells

Quantification of circulating endothelial progenitor cells (EPCs) and mature endothelial cells (ECs) were quantified according to Duda et al. (23), with minor modifications. The antibody panel included CD45-PerCP, CD61-FITC, CD34-FITC, CD133-PE, CD184-APC (CXCR4), and KDR-APC (VEGFR-2). EPCs were defined as CD45^-^/CD61^-^/CD133^+^/CD34^+^/CD184^+^/KDR^+^, and ECs as CD45^-^/CD61^-^/CD133^-^/CD34^+^/CD184^+^/KDR^+^. At least 5 × 10^5^ events were recorded per sample. Cells were stained at 4 °C for 30 minutes in the dark, washed, and resuspended in PBS before acquisition.

### 2.8 Statistical analysis

Statistical analyses were performed using R software (v4.5.0). Data are expressed as mean ± standard error of the mean (SEM). Paired comparisons (pre- vs. post-treatment) were evaluated using two-tailed paired t-tests. No correction for multiple testing was applied due to the exploratory nature of the study. A p-value <0.001 was considered statistically significant. When applicable, multivariate analyses, including linear regression, discriminant analysis, and principal component analysis, were employed to identify predictors of biological response. Normality of paired differences was assessed with Shapiro–Wilk. Paired t-tests were applied when assumptions were satisfied; otherwise, Wilcoxon signed-rank tests were used. Effect sizes (Cohen’s d or matched-pairs rank-biserial correlation) and 95% confidence intervals are now reported for all outcomes. As a sensitivity analysis for multiple testing across the predefined biomarker family (n = 18), paired-test p-values were adjusted using the Benjamini–Hochberg false discovery rate (FDR) and Holm–Bonferroni procedures. For values reported as < 0.0001, a conservative p = 0.0001 was applied prior to adjustment. Adjusted p-values are presented in **Supplementary Table S1**.

### 2.9 Safety monitoring

Safety analyses entailed the evaluation of all adverse reactions, especially severe or unforeseen occurrences, and treatment terminations. Case report forms (CRFs) were individually reviewed for completeness and protocol adherence. CRFs with severe inconsistencies or incomplete data were excluded.

### 2.10 Ethical considerations

This study was conducted in accordance with the principles of the Declaration of Helsinki and complied with local regulations governing retrospective studies involving human subjects. The protocol was approved by the Ethics Committee of Hospital Universitario Virgen de la Victoria, Málaga (SMD-STRESS01-2008). No identifiable personal data were used in the analysis.

## 3 Results

All reported p-values were also subjected to multiplicity correction using Benjamini–Hochberg FDR and Holm–Bonferroni procedures (see **Supplementary Table S1**). All major signals remained significant after adjustment, confirming the robustness of the findings.

### 3.1 Reduction of iron overload

Serum ferritin levels were measured in 23 patients with MDS before and after treatment with deferasirox to evaluate the efficacy of iron chelation in reducing systemic iron burden. All patients had elevated baseline ferritin levels, consistent with chronic transfusion dependency (mean ± SEM: 2090.87 ± 148.38 ng/mL), reflecting clinically relevant iron overload. A statistically significant reduction in serum ferritin was observed after chelation therapy, with mean post-treatment levels dropping to 946.39 ± 14.51 ng/mL (p<0.0001). This reduction corresponds to an approximate 55% decrease in systemic iron burden, highlighting the physiological efficacy of iron chelator in mitigating transfusion-induced iron accumulation. The study did not specifically record transfusion burden data, but all patients had received 20 or more red blood cell units in the year before being included, thus meeting the threshold for significant clinical iron overload. A detailed summary of the biological parameters analysed before and after chelation is provided in **Supplementary Table S2**.

### 3.2 Effect of iron chelation on vascular damage and endothelial activation in patients with MDS

**Figure 1:**
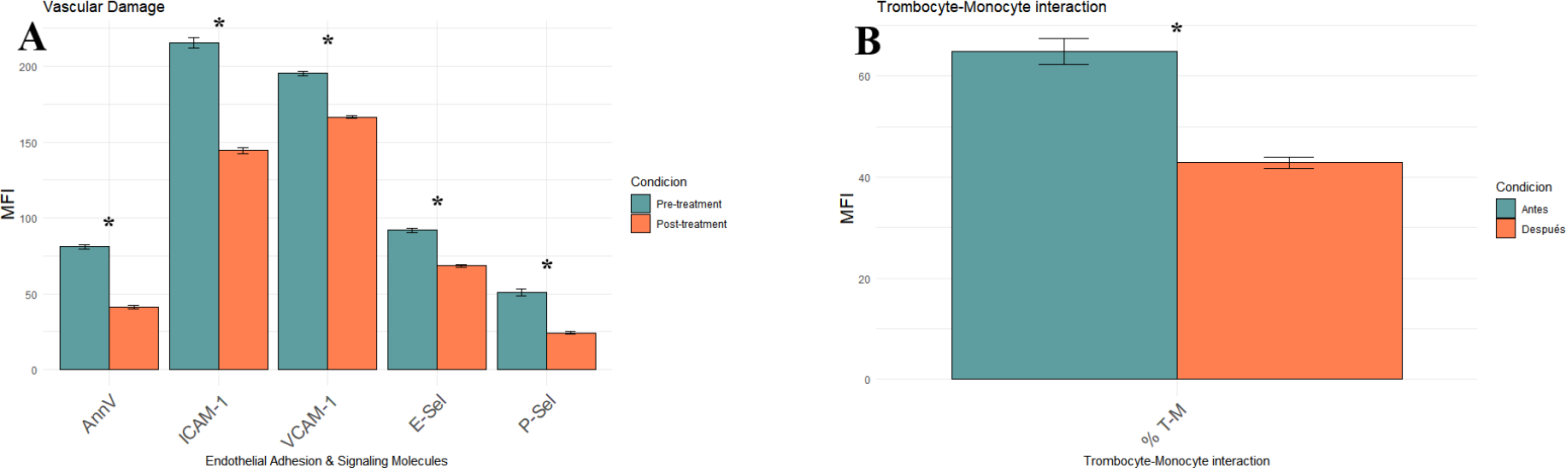
Effect of iron chelation on endothelial activation and thromboinflammatory markers in MDS patients, comparing pre- (green) and post-treatment (orange) groups. **(A)** Bar plot showing mean MFI ± SEM for five vascular injury markers: Annexin V, ICAM-1, VCAM-1, E-selectin (E-sel) and P-selectin (P-sel). **(B)** Bar plot displaying the percentage of monocyte–platelet aggregates (% T–M). All values represent the mean of 23 patients ± SEM. Statistical significance was determined using paired t-tests. Asterisks (*) p-values < 0.0001.

We quantified six surface markers related to endothelial injury, adhesion molecule expression, and thromboinflammatory activation to explore the impact of iron overload on vascular homeostasis in MDS. All parameters were measured by flow cytometry and are expressed as mean fluorescence intensity (MFI). Baseline Annexin V expression was significantly elevated (81.00 ± 1.5), indicating increased apoptotic activity and cellular stress in the hematopoietic-endothelial compartment (**Figure 1A**). Following iron chelation, Annexin V levels were significantly reduced to 41.13 ± 1.03 MFI (p<0.0001), indicating a substantial attenuation of apoptotic signaling. Endothelial adhesion markers were significantly downregulated following treatment: ICAM-1, decreased from 215.35 ± 3.36 to 144.43 ± 1.86 MFI, VCAM-1, from 195.17 ± 1.51 to 166.52 ± 0.95 MFI (both p<0.0001). Iron chelation led to downregulation of E-selectin (91.87 ± 1.28 to 68.51 ± 0.74 MFI) and P-selectin (50.96 ± 2.17 to 24.26 ± 0.89 MFI), key mediator of leukocyte and platelet vascular adhesion. These reductions were also statistically significant (p<0.0001), reinforcing the hypothesis that iron overload intensifies endothelial adhesiveness and promotes a proinflammatory vascular phenotype. The interaction between T cells and monocytes (as depicted in **Figure 1B**), measured as the percentage of monocytes forming aggregates with platelets—a marker of thromboinflammatory response—dropped significantly from 64.87% ± 2.64 to 42.87% ± 1.15 after the treatment (p<0.0001; see dot plots in **Supplementary Figures S1D–E**).

### 3.3 Effect on endothelial progenitor cells

**Figure 2:**
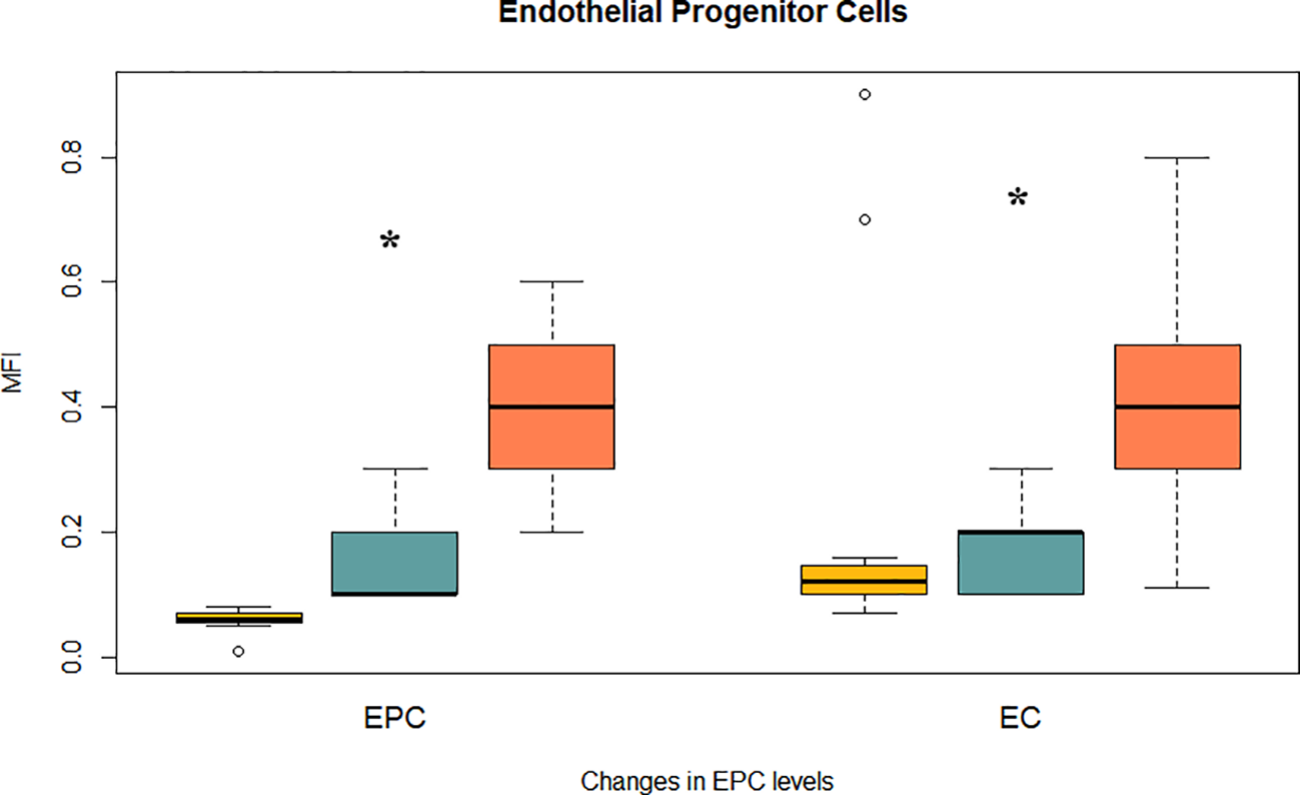
Effect of iron chelation on endothelial damage and progenitor cells. Bar plots show mean MFI ± SEM for two vascular regenerative populations assessed by flow cytometry: Endothelial progenitor cells (EPCs): CD45^-^/CD61^-^/CD133^+^/CD34^+^/CD184^+^/KDR^+^; and Mature endothelial cells (ECs): CD133^-^/CD34^+^/CD184^+^/KDR^+^ (with identical background markers). Data are shown for healthy controls (yellow), MDS patients before chelation (blue), and after treatment (orange). (*) indicate statistically significant differences between treatment groups (p < 0.0001).

To determine whether iron chelation improves vascular regenerative capacity, we evaluated the levels of circulating endothelial progenitor cells (EPCs) and mature endothelial cells (ECs) by flow cytometry. EPCs were defined as CD45^-^/CD61^-^/CD133^+^/CD34^+^/CD184^+^/KDR^+^ cells, and ECs as CD133^-^ with the same remaining markers. At baseline, MDS patients exhibited reduced EPC and EC levels, consistent with impaired endothelial turnover. Iron chelator significantly increased both EPCs (0.17 ± 0.02 to 0.41 ± 0.02 MFI) and ECs (0.17 ± 0.01 to 0.43 ± 0.03 MFI; both p < 0.0001). Importantly, post-treatment values for both EPCs and ECs not only recovered but surpassed those observed in healthy controls (EPCs: 0.06 ± 0.004; ECs: 0.30 ± 0.078; **Figure 2**).

### 3.4 Iron chelation and mitochondrial function

**Figure 3:**
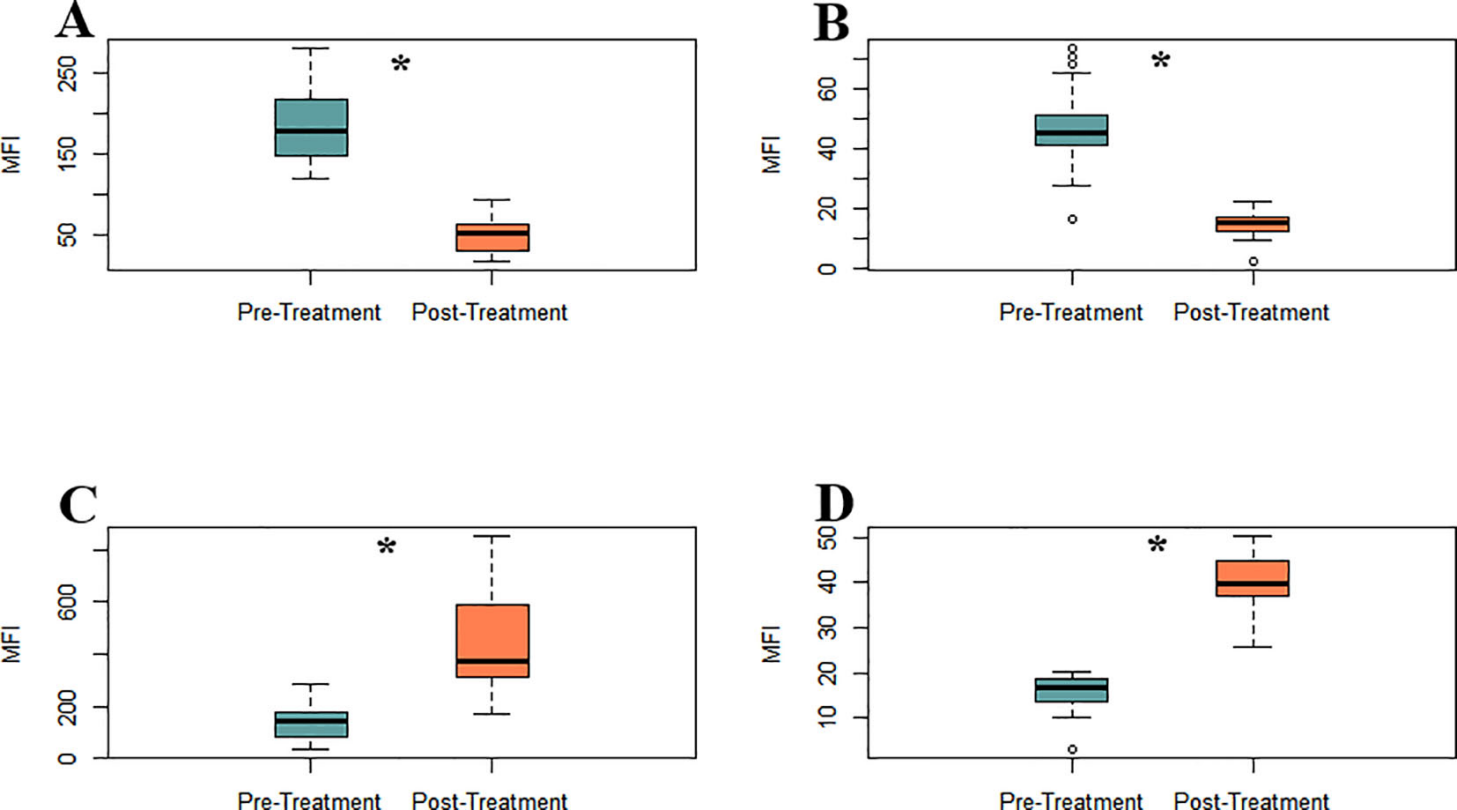
Iron chelation reduces oxidative stress and restores mitochondrial membrane potential in MDS leukocytes between pre- (green) and post-treatment (orange) groups. **(A)** H_2_O_2_ levels. **(B)** O_2_^-^ levels **(C)** Intracellular glutathione levels. **(D)** Mitochondrial membrane potential, assessed by Rhodamine-123 staining, Asterisks (*) indicate statistically significant differences between treatment groups (p < 0.0001).

To explore the pathophysiological impact of iron overload on redox homeostasis and mitochondrial integrity in MDS, a comprehensive cytometric assessment of oxidative stress was conducted. Parameters and mitochondrial membrane potential in leukocyte populations were analysed before and after iron chelation treatment. Pre-treatment ROS levels were markedly reduced, specifically, hydrogen peroxide (H_2_O_2_, 185.08 ± 10.19 vs. 49.31 ± 4.36 MFI, as well as superoxide (O_2_^-^, 47.43 ± 2.79 vs. 14.87 ± 0.92 MFI; both p < 0.0001; **Figure 3B**). Thus, a robust upregulation of endogenous antioxidant defenses (**Figure 3C**; **Supplementary Figures S1B, C**), as evidenced by a marked elevation in intracellular glutathione levels post-treatment (136.03 ± 13.36 vs. 445.51 ± 40.05 MFI; p < 0.0001) was observed. Mitochondrial polarization was evaluated using Rhodamine-123 staining as a surrogate for membrane potential. Following chelation, leukocytes exhibited a significant increase in membrane potential MFI (15.58 ± 0.84 vs. 39.84 ± 1.42 MFI; p < 0.0001), indicative of improved mitochondrial function (**Figure 3D**; **Supplementary Figures S1A, F**). **Supplementary Figure S1** provides representative flow cytometry plots illustrating the observed changes in mitochondrial potential, oxidative stress, intracellular glutathione levels, and monocyte–platelet aggregates.

### 3.5 Pro-inflammatory interleukins and clinical status

**Figure 4:**
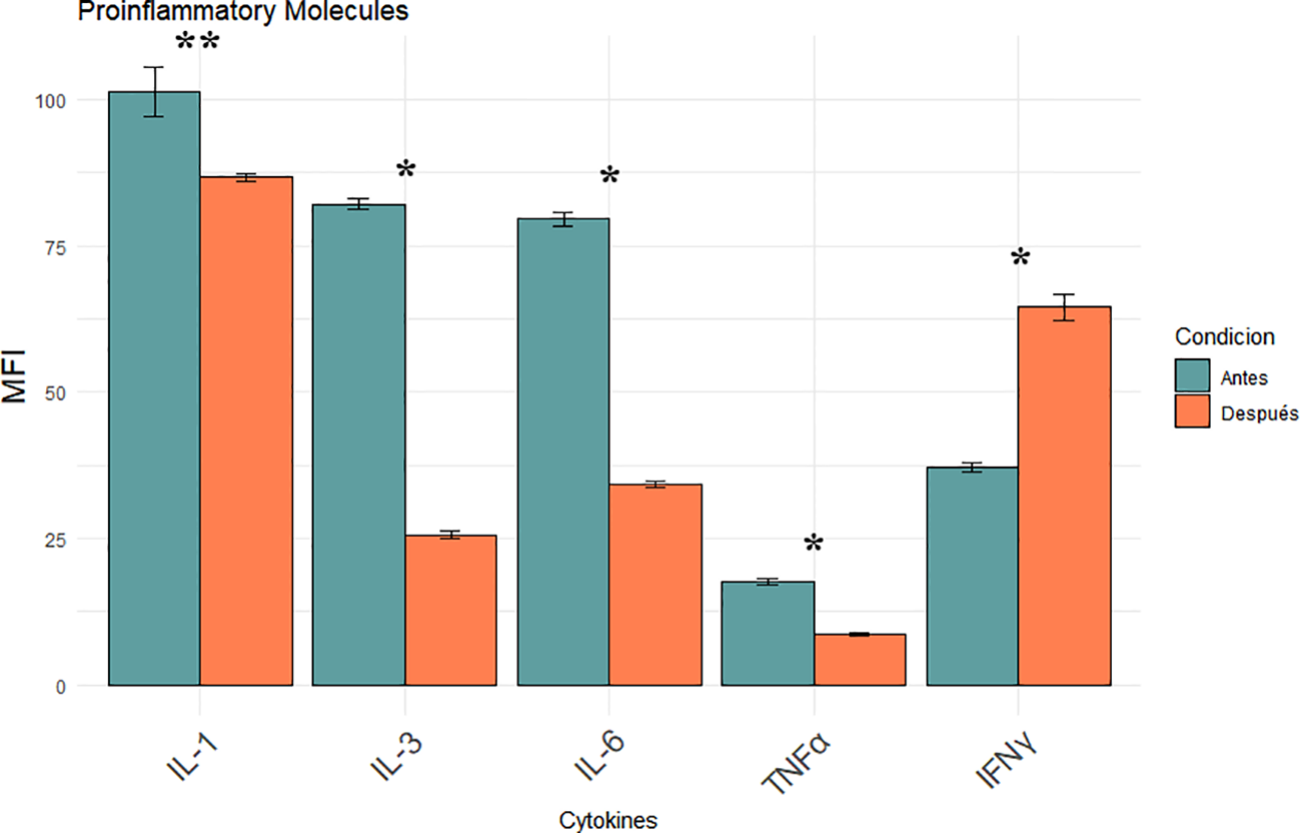
Modulation of pro-inflammatory cytokines (IL-1, IL-3, IL-6, TNF- α, IFN-γ) following iron chelation in MDS patients. Bar plots show the mean MFI ± SEM of five key cytokines measured in leukocytes by flow cytometry before (blue) and after (orange) iron chelator treatment. Asterisks indicate statistically significant differences between pre- and post-treatment groups ([*] p < 0.0001, [**] p = 0.0013).

Pro-inflammatory cytokines were analysed to evaluate the impact of iron chelation on the inflammatory milieu in MDS before and after iron chelator treatment. The panel included interleukin-1 (IL-1), interleukin-3 (IL-3), interleukin-6 (IL-6), tumor necrosis factor-alpha (TNF-α), and interferon-gamma (IFN-γ), all measured by flow cytometry and expressed as mean MFI ± SD. Post-treatment analysis revealed a significant reduction in IL-1 expression (101.22 ± 4.27 vs. 86.70 ± 0.66 MFI; p = 0.0013) indicating partial suppression of inflammasome-driven signaling (**Figure 4**). More pronounced decreases were observed for IL-3 and IL-6, both key regulators of hematopoietic proliferation and inflammatory skewing in MDS, with levels dropping to 82.13 ± 1.02 to 25.65 ± 0.68 and 79.52 ± 1.31 to 34.22 ± 0.57 MFI respectively (p < 0.0001). TNF-α levels were also significantly reduced (17.52 ± 0.50 to 8.70 ± 0.33 MFI; p < 0.0001). suggesting reduced activation of the monocyte-macrophage axes., (**Figure 4**). Conversely, IFN-γ expression increased significantly after treatment (37.22 ± 0.81 to 64.52 ± 2.19 MFI; p < 0.0001). No significant changes were observed in classical hematological parameters, nor were any correlations found between these variables and the biological endpoints assessed, pointing to a predominantly immunomodulatory effect of iron chelation therapy.

## 4 Discussion

**Figure 5:**
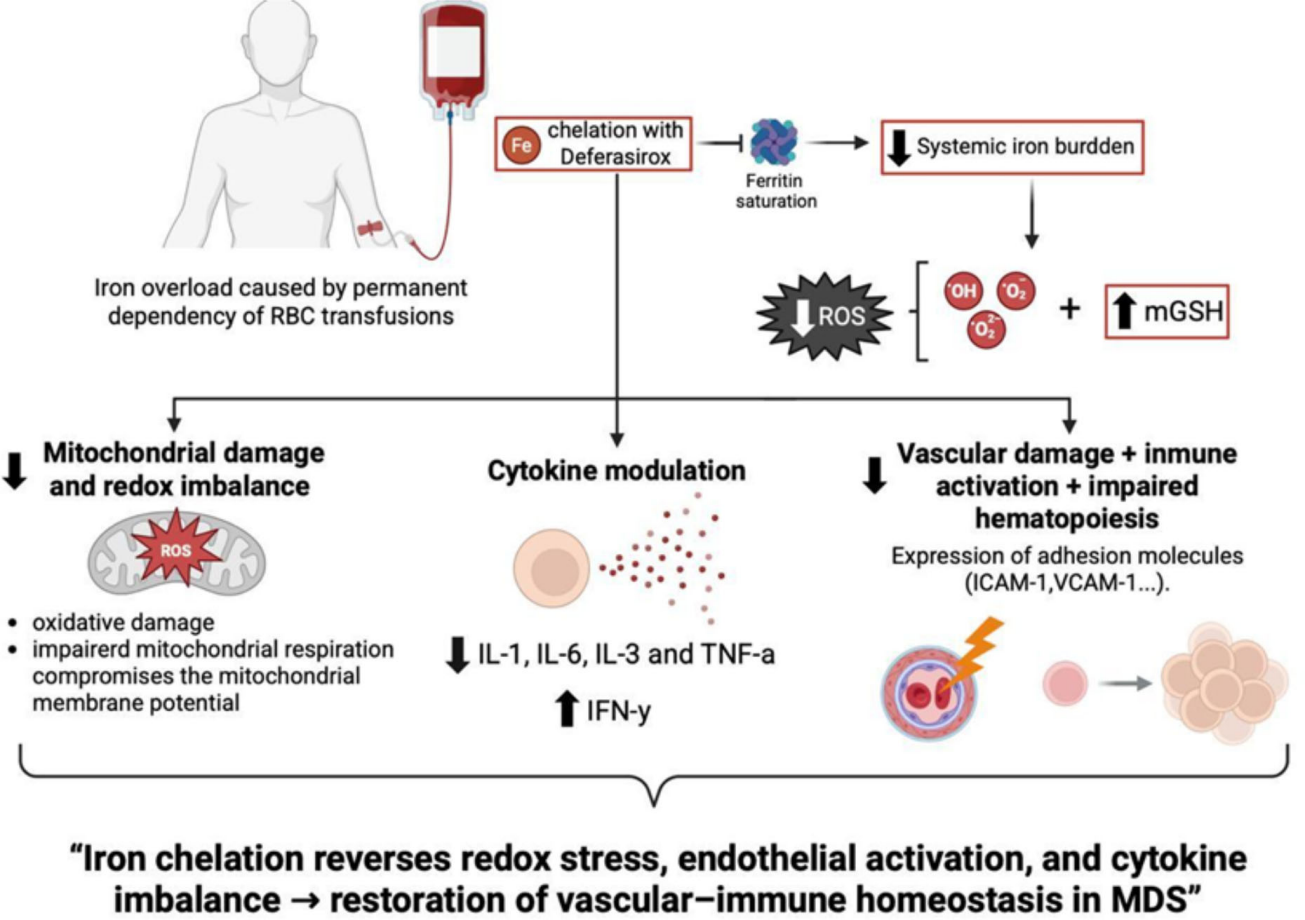
Conceptual model linking iron overload to mitochondrial dysfunction, endothelial activation, and inflammation in MDS, and its reversal by iron chelation therapy. Iron accumulation triggers ROS generation, glutathione depletion, and mitochondrial damage, leading to MAPK-driven cytokine production and vascular inflammation. Deferasirox restores redox balance and mitigates these effects, contributing to improved hematopoietic homeostasis.

Chronic transfusion dependence is a hallmark of advanced myelodysplastic syndrome (MDS), leading to iron accumulation in the system. Multiple studies have identified elevated serum ferritin as an independent adverse prognostic factor in MDS, with levels ≥500–1000ng/mL correlated with reduced OS, irrespective of transfusion frequency (24, 25). Our longitudinal, paired-sample study was designed as a mechanistic, hypothesis−generating investigation. We acknowledge the single-center setting, modest cohort size (n=23), and historical enrolment as limitations for external validity, and we encourage prospective multicenter studies to validate and expand these observations. This cohort showed a robust and homogeneous reduction of approximately 55% in mean ferritin levels following daily oral deferasirox therapy, indicating effective control of systemic iron burden, including patients who had received heavy transfusions. Iron functions not only as an indicator of transfusional toxicity, but also as a redox-active metal that accelerates the Fenton reaction, producing reactive oxygen species (ROS) that cause damage to DNA, mitochondrial membranes, and components of the hematopoietic microenvironment. This oxidative stress may contribute to genomic instability, hematopoietic stem cell (HSC) dysfunction, clonal hematopoiesis progression, and a propensity to develop acute myeloid leukemia (AML) (6, 26).Our data suggest that the observed reduction in ferritin may contribute to improvements in redox status, mitochondrial potential, endothelial function, and cytokine profile, supporting the role of iron chelation in biological recovery. Furthermore, clinical studies have positioned iron chelation as a potential disease-modifying intervention in lower-risk MDS. Retrospective analyses and registry data have demonstrated improved overall survival and hematologic responses in patients with chelation, particularly when initiated early and maintained consistently (27, 28). These observations align with our data and reinforce the concept of targeting iron metabolism as a strategic entry point into modifying the disease course. Iron overload induces endothelial activation and dysfunction *via* non-transferrin-bound iron (NTBI)–mediated oxidative stress, promoting leukocyte and platelet adhesion to the endothelium (29). Deferasirox treatment significantly reduced endothelial apoptosis (Annexin V), adhesion molecules (ICAM-1, VCAM-1), and selecting (Eselectin, Pselectin) and decreased monocyteplatelet aggregates (T–M interaction) (**Figures 1A, B**). These findings are consistent with preclinical data showing that NTBI directly induces endothelial injury and upregulates adhesion molecule expression, a process reversible with iron chelation (30). Elevated ICAM1 and VCAM1 levels are considered hallmarks of a pro-inflammatory endothelium and contribute to vascular remodeling and atherosclerosis (31). Their reduction suggests endothelial normalization, which is further supported by the increase in circulating endothelial progenitor cells (EPCs) and mature endothelial cells (ECs) (**Figure 2**). EPCs are essential for vascular repair and angiogenesis (32), and prior studies in murine models indicate that impaired EPC function exacerbates MDS-related endothelial dysfunction (8).Our observation that EPC and EC levels recovered post-chelation strongly supports the hypothesis that iron detoxification promotes vascular regeneration in MDS. Excess intracellular iron not only impacts endothelial cells but also induces leukocyte oxidative stress, disrupting mitochondrial function (29). In our cohort, iron chelators reduced leukocyte hydrogen peroxide and superoxide levels (**Figures 3A, B**) and restored intracellular glutathione (**Figure 3C**), supporting the recovery of redox balance. This is consistent with the central role of iron-catalyzed ROS production in hematopoietic dysfunction (33). Furthermore, the observed increase in mitochondrial membrane potential (**Figure 3D**) suggests recovery of mitochondrial integrity—a critical aspect given that mitochondrial dysfunction contributes to hematopoietic failure and clonal evolution in MDS (34). Together, these data demonstrate that iron chelation supports not only antioxidant recovery but also bioenergetic reactivation at the cellular level. Deferasirox administration resulted in significant reductions in IL1, IL3, IL6, and TNF-α, pivotal mediators of chronic inflammation and dysregulated hematopoiesis in MDS (**Figure 4**). This cytokine suppression aligns with established models whereby iron overload enhances pro-inflammatory signaling *via* NTBI-induced myeloid activation and marrow microenvironment perturbation (35, 36). Of particular interest, IFN-γ levels rose following treatment, implying a potential recalibration toward enhanced immune surveillance rather than a mere pro-inflammatory rebound (37). This cytokine modulation suggests that iron chelation exerts a dual immunological impact: it downregulates pathogenic myeloid inflammatory circuits and may simultaneously restore adaptive immune competence. Elevated IL-6 and TNF-α in MDS have been linked to STAT3-driven clonal survival, increased proliferation, and epigenetic alterations (38); therefore, their decline could mitigate oncogenic selective pressure. Conversely, increased IFN-γ is known to enhance antigen presentation and apoptotic susceptibility in malignant progenitors (39), supporting a paradigm in which iron removal promotes not only detoxification but also immunologic rejuvenation of hematopoiesis, which ultimately translates into a reduction in the progression of these patients to AML. Limitations of this study should be acknowledged. First, although the internal consistency of the biological signals observed is strong, the absence of a comparator group—such as patients receiving alternative iron chelators or no chelation—precludes conclusions about specificity. Second, the study was not designed to assess clinical outcomes such as hematologic response, progression to acute leukemia, or overall survival. Finally, no single biomarker was prespecified as a primary outcome, since our intention was to interrogate coordinated changes across mechanistic axes rather than to test a clinical endpoint. While multiple parameters were analysed, the strength of our findings derives from the internal consistency of changes observed across related biomarkers, rather than isolated p-values. For this reason, formal multiplicity corrections were not applied, as they are more appropriate in confirmatory clinical trials than in exploratory mechanistic studies such as ours. While the present data provide mechanistic insights into the biological effects of iron chelation, future studies integrating clinical endpoints will be necessary to validate the translational impact of these findings. The cytokine measurements were based on MFI *via* flow cytometry rather than absolute quantification in plasma. Despite this, the consistency of directional trends across multiple cytokines, in concert with simultaneous reductions in ferritin and oxidative stress, strongly supports a model in which iron chelation actively remodels the inflammatory milieu to favor hematopoietic equilibrium. In sum, these findings position iron chelation as a multifaceted intervention—simultaneously vascular, metabolic, and immunologic—capable promoting biological re-equilibration of MDS pathobiology towards a more homeostatic state and justifying further mechanistic and clinical validation. Collectively, the findings presented here support a unifying biological model in which transfusion-related iron overload induces a cascade of mitochondrial dysfunction, oxidative stress, endothelial activation, and inflammatory skewing. Iron chelation reverses these events by restoring redox homeostasis and modulating immune-endothelial crosstalk. This integrative framework is summarized in **Figure 5** and offers a mechanistic rationale for positioning iron chelator as a disease-modifying agent in MDS. While these results are mechanistic and hypothesis-generating rather than definitive evidence of disease modification, they provide a biologically coherent rationale for prospective studies integrating clinical endpoints with immune-metabolic readouts.

## Data Availability

The original contributions presented in the study are included in the article/**Supplementary Material**. Further inquiries can be directed to the corresponding authors.

## Supplementary material

Supplementary Figure 1 Representative flow cytometry plots showing adhesion molecules, oxidative stress, mitochondrial membrane potential, and glutathione levels.

Supplementary Table 1 Multiplicity-adjusted p-values for all biomarkers analysed in transfusion-dependent MDS patients.

Supplementary Table 2 Summary of biological parameters analysed before and after iron chelation therapy.
