# Supplementary material for: Impact of iron chelation therapy on mitochondrial function, vascular integrity and inflammation in transfusion-dependent myelodysplastic syndromes
Source: Front Immunol. 2025 Nov 10;16:1683941. doi: 10.3389/fimmu.2025.1683941 (PMC12640865; doi:10.3389/fimmu.2025.1683941)
Supplement: Supplementary Figure 1 — Representative flow cytometry plots showing adhesion molecules, oxidative stress, mitochondrial membrane potential, and glutathione levels. [file SupplementaryFile1.docx]

| 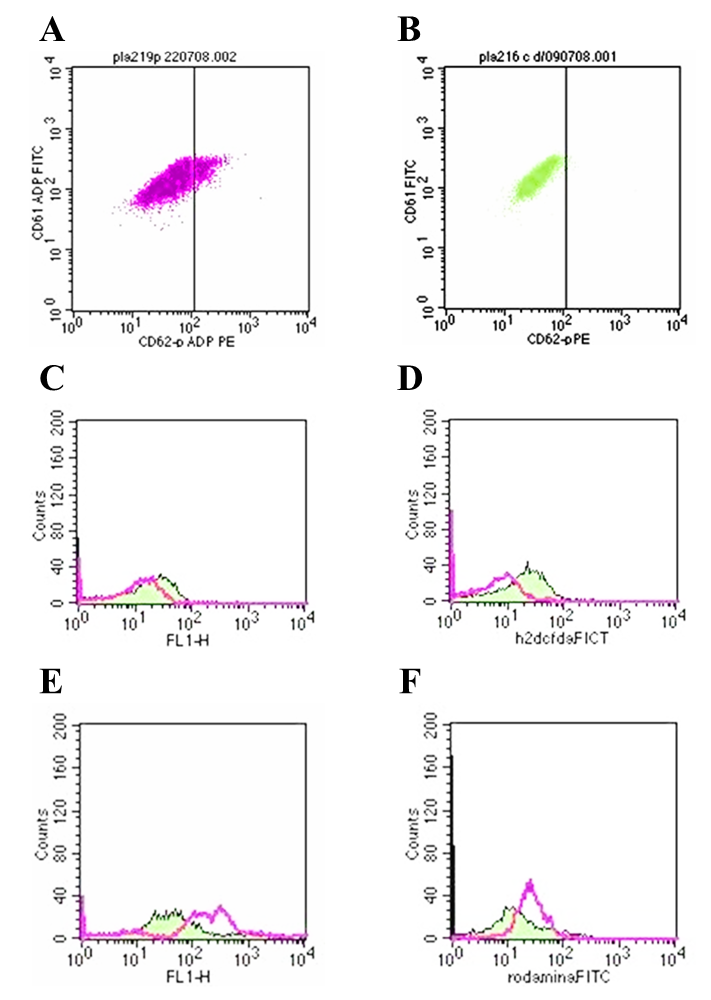 |
| --- |
| **Figure S1.** Representative flow cytometry plots illustrating endothelial adhesion, oxidative stress, antioxidant status, and mitochondrial function in MDS patients before and after iron chelation therapy. (A–B) Dot plots of CD61-FITC versus CD62p-PE showing the reduction in monocyte–platelet aggregates and selectin expression after treatment. (C–D) Overlay histograms of intracellular reactive oxygen species (ROS) measured by FL1-H channel using H₂DCFDA. (E) Intracellular glutathione levels (mGSH) assessed by monochlorobimane fluorescence (mBCI). (F) Mitochondrial membrane potential measured by Rhodamine-123 staining (FITC channel). Green: pre-treatment; Magenta: post-treatment. |
