# Supplementary material for: Impact of iron chelation therapy on mitochondrial function, vascular integrity and inflammation in transfusion-dependent myelodysplastic syndromes
Source: Front Immunol. 2025 Nov 10;16:1683941. doi: 10.3389/fimmu.2025.1683941 (PMC12640865; doi:10.3389/fimmu.2025.1683941)
Supplement: Supplementary Table 1 — Multiplicity-adjusted p-values for all biomarkers analysed in transfusion-dependent MDS patients. [file SupplementaryFile2.docx]

**Supplementary Table S1.** Sensitivity analysis for multiplicity across the predefined biomarker family (n = 18 outcomes). Reported paired-test p-values were adjusted using Benjamini–Hochberg FDR and Holm–Bonferroni procedures. For values reported as “<0.0001”, a conservative p = 0.0001 was used for adjustment. All major signals remained significant after correction.

| Outcome | Raw p-value (reported) | Numeric p used for adj. | BH-FDR q-value | Holm–Bonferroni p_adj |
| --- | --- | --- | --- | --- |
| Serum Ferritin (ng/mL) | <0.0001 | 0.0001 | 0.000556 | 0.0018 |
| Annexin V (MFI) | <0.0001 | 0.0001 | 0.001111 | 0.0017 |
| ICAM-1 (MFI) | <0.0001 | 0.0001 | 0.001667 | 0.0016 |
| VCAM-1 (MFI) | <0.0001 | 0.0001 | 0.002222 | 0.0015 |
| E-selectin (MFI) | <0.0001 | 0.0001 | 0.002778 | 0.0014 |
| P-selectin (MFI) | <0.0001 | 0.0001 | 0.003333 | 0.0013 |
| Monocyte-platelet aggregates (T–M interaction, MFI) | <0.0001 | 0.0001 | 0.003889 | 0.0012 |
| EPC (flow readout as reported) | <0.0001 | 0.0001 | 0.004444 | 0.0011 |
| EC (flow readout as reported) | <0.0001 | 0.0001 | 0.005 | 0.001 |
| Leukocyte H2O2 (MFI) | <0.0001 | 0.0001 | 0.005556 | 0.0009 |
| Leukocyte O2− (MFI) | <0.0001 | 0.0001 | 0.006111 | 0.0008 |
| Leukocyte Glutathione (MFI) | <0.0001 | 0.0001 | 0.006667 | 0.0007 |
| Mitochondrial Membrane Potential ΔΨm (MFI) | <0.0001 | 0.0001 | 0.007222 | 0.0006 |
| IL-1 (MFI) | 0.0013 | 0.0013 | 0.0013 | 0.0013 |
| IL-3 (MFI) | <0.0001 | 0.0001 | 0.008333 | 0.0004 |
| IL-6 (MFI) | <0.0001 | 0.0001 | 0.008889 | 0.0003 |
| TNF-α (MFI) | <0.0001 | 0.0001 | 0.009444 | 0.0002 |
| IFN-γ (MFI) | <0.0001 | 0.0001 | 0.01 | 0.0001 |
