# Supplementary material for: Impact of iron chelation therapy on mitochondrial function, vascular integrity and inflammation in transfusion-dependent myelodysplastic syndromes
Source: Front Immunol. 2025 Nov 10;16:1683941. doi: 10.3389/fimmu.2025.1683941 (PMC12640865; doi:10.3389/fimmu.2025.1683941)
Supplement: Supplementary Table 2 — Summary of biological parameters analysed before and after iron chelation therapy. [file SupplementaryFile3.docx]

**Table S1.** Summary of measurements of data collected before and after treatment with iron chelators (n=23)

| Variables | Before Treatment | After Treatment |
| --- | --- | --- |
| Serum Ferritin (ng/mL) | 2090,87 ± 148,38 | 946,39 ± 14,51 |
| *Vascular Damage Markers* | | |
| ****Annexin V**** (MFI) | 81,00 ± 1,5 | 41,13 ± 1,03 * |
| ICAM-1 (MFI) | 215,35 ± 3,36 | 144,43 ± 1,86 * |
| VCAM-1 (MFI) | 195,17 ± 1,51 | 166,52 ± 0,95 * |
| E-selectin (MFI) | 91,87 ± 1,28 | 68,51 ± 0,95 ***** |
| P-selectin (MFI) | 50,96 ± 2,17 | 24,26 ± 0,89 * |
| Percentaje T-M (MFI) | 64,87 ± 2,64 | 42,87 ± 1,15 ***** |
| *Endothelial Progenitor Cells* | | |
| EPC (MFI) | 0,17 ± 0,02 | 0,41 ± 0,02 ***** |
| EC (MFI) | 0,17 ± 0,01 | 0,43 ± 0,03 ***** |
| *Oxidative Stress Markers* | | |
| Leukocyte H peroxide (MFI) | 185,08 ± 10,19 | 49,31 ± 4,36 ***** |
| Leukocyte O peroxide (MFI) | 47,43 ± 2,79 | 14,87 ± 0,92 * |
| Leukocyte glutathione (MFI) | 136,03 ± 13,36 | 445,51 ± 40,05 * |
| *Mitochondrial Function* | | |
| Mitochondrial Membrane Potencial (MFI) | 15,58 ± 0,84 | 39,84 ± 1,42 * |
| *Pro-inflammatory Cytokines* | | |
| IL-1 (MFI) | 101,22 ± 4,27 | 86,70 ± 0,66 ** |
| IL-3 (MFI) | 82,13 ± 1,02 | 25,65 ± 0,68 * |
| IL-6 (MFI) | 79,52 ± 1,31 | 34,22 ± 0,57 * |
| TNF-α (MFI) | 17,52 ± 0,5 | 8,7 ± 0,33 * |
| IFN-γ (MFI) | 37,22 ± 0,81 | 64,52 ± 2,19 ***** |
| ** Statistically significant difference (p < 0,0001)* | | |
| *** Statistically significant difference (p = 0,0013)* | | |

Summary of biological parameters in MDS patients before and after iron chelation.

Data are expressed as mean ± SEM. Statistical significance was assessed using paired t-tests. *p < 0.0001; **p = 0.0013.
